# The Book World of Medicine and Science

**Published:** 1894-06-16

**Authors:** 


### The Book World of Medicine and Science.

Central Blatt für Chirurgie. (April, 1894.)

In this number Dr. Felix Loewenhardt, of Breslau, gives an account of a new method of endoscoping the male urethia. The ordinary methods at present in use depend on a principle of illumination from the exterior, the light, by a series of reflectors, being thrown down the endoscope tube. This precludes the satisfactory employment of magnifying lenses within the tube. Dr. Loewenhardt employs illumination from the bladder, which can be filled with water and incandescent lamp thus kept cool. The method is highly ingenious and has obvious applications for other viscera. Dr. Fredrik Ramm, of Christiana, suggests the treatment of hypertrophied prostate by castration, the inter-association of growth and development between the testes and prostate is such that the removal of the former is closely followed by the involution of the latter. His statement, however, seems rather opposed to the known fact that in old age, when the prostate hypertrophies, the testes are in process of anatomical and physiological atrophy.

Zeitschrift für Hygiene und Infectionskrankheilen. (Leipsig, April, 1894.)

In this number of the " Zeitschrift " Drs. Claudio Fermi and Leone Pernossi publish a complete account of the poison of tetanus. The laborious series of experiments by which they attempt to establish the nature of the virus is given in extenso, and without doubt this is the most complete and accurate history of the poison which has appeared up to the present date. In a very useful summary at the end of their paper the authors give a comparative table of the properties of the poisons of tetanus-diphtheria naja tripudians, &c. The points to which they pay especial attention are the influence of heat, light, and chemical reagents on the virus. They also describe the influence of the intestinal secretions on the poison, the rapidity of absorbtion, the smallest dose, the susceptibility of various animals, and the chemical nature of the active principle as given by various authorities. The most important conclusions at which the authors arrive are as follows: (1) The tetanus poison is neither a ferment, nor has it any relationship with ferments. (2) It resembles a coltoid substance rather than a non-coltoid, and an albuminoid rather than a non-albuminoid. (3) The only solvent is water. In the same number Dr. R. Stern gives some interesting facts relating to the effect of the blood of typhoid patients, when injected into animals, causing immunity from typhoid symptoms when the typhoid bacillus is subsequently injected. Professor H. Leo and Dr. R. Sondumann give a comprehensive account of the biology of the cholera baccillus.

